# Environmental DNA transformation resulted in an active phage in *Escherichia coli*

**DOI:** 10.1371/journal.pone.0292933

**Published:** 2023-10-13

**Authors:** Abdulkerim Karaynir, Bülent Bozdoğan, Hanife Salih Doğan

**Affiliations:** 1 Recombinant DNA and Recombinant Protein Center (REDPROM), Aydın Adnan Menderes University, Aydın, Türkiye; 2 Medical Faculty, Department of Medical Microbiology, Aydın Adnan Menderes University, Aydın, Türkiye; University of Jeddah, SAUDI ARABIA

## Abstract

The achievement of an active biological entity from environmental DNA is important in the field of phage. In this study, the environmental DNA extracted from hospital wastewater was transferred into *Escherichia coli* DH10B and *Escherichia coli* BL21 with chemical transformation and electroporation. After transformation, overnight cultures were filtered and used as phage source. The efficacies of the techniques were evaluated with spot test and double-layer agar assay. The emerged phage, named as ADUt, was purified and host-range analysis was performed. Phage DNA was isolated, sequenced and restriction profile was determined. The genome was assembled. The phylogenetic tree was constructed via VipTree. The extracted DNA resulted in active phage by the transformation of *E*. *coli* DH10B, but not *E*. *coli* BL21. The chemical transformation was found more successful than electroporation. ADUt phage was found to be polyvalent and effective against limited strains of *Shigella* and *Escherichia* genera. The phage genome size and GC ratio are 166904 bp and 35.67%, respectively. ADUt is a member of *Straboviridae* family and *Tequatrovirus* genus. This is the first study that uses environmental DNA for acquiring active phage, which may be an important source of new phage discovery. The result showed that DNA transformation yields active bacteriophage with both chemical transformation and electroporation.

## Introduction

Bacteriophage is one of the mediators of discovery of DNA as genetic material. Hershey-Chase experiment is one of justifiers for that Griffith’s “transforming principle”. In the beginning of 20^th^ century, Griffith’s revealed a molecule in bacterium that is responsible for inheritance of genetic information. Avery-MacLeod-McCarthy and Hershey-Chase proved “transforming principle”. Avery-MacLeod-McCarthy used bacteria, while Hershey-Chase used bacteriophages to demonstrate DNA’s heredity amenability, not a protein [1]. Based on the scientific literature contribution of bacteriophages, they are also valuable warriors against infections. In contrast to popularity of antibiotics for roughly 50 years, bacteriophages have been discovered earlier by Tworth and d’Herelle in 1915 and 1917, respectively, as regards d’Herelle’s statement [2]. The phage therapy that has begun with treatment of patient suffered from dysentery by d’Herelle, is still proceeding to treat various infections. The important advances about bacteriophages were interrupted by World War I. In addition to financial limitations because of war, discovery of antibiotics decreased attention and investments to phage research [3]. However, the phage research and application in clinical has lasted until these days in some countries of former Soviet Union [4]. The most famous one is George Eliava Institute of Bacteriophages in Georgia [5]. Nowadays, Europe and US are refocusing on phage therapy to overcome “superbugs” [4, 6].

Bacteriophages have been used quite widely including; the therapeutic and prophylactic purposes, protection of plants from infectious agents and to hold foods in safe [7–9]. The innovative usage of bacteriophages is phage display that facilitates display short peptides or antibodies on its surface [10].

The phages are found in various places worldwide including soil, ocean, river, wastewater, and gut etc. The phages that are obtained from these places can be used directly or indirectly against bacteria or the lytic enzymes encoded by the phages can be used against bacteria [11]. Some of the phages and their hosts found in these locations can be cultured hardly or cannot be cultured in laboratory conditions. To overcome these challenges, transformation method can be an alternative option. Transformation of bacteria with known and isolated phages genome has been processed since 1968. Firstly, T4 bacteriophage DNA was transferred to *E*. *coli* after spheroplast formation and the multiplication of phages were observed [12]. In time, transformation of different bacteria species such as *Staphylococcus aureus*, *Pseudomonas aeruginosa* etc. were reported with various phage DNA [13, 14]. Nowadays, the transformation is used to transplantation of genetically engineered phage genome into various bacteria [15–17].

Differently from previous studies, the transformation of phage DNA was used for different purpose. In this study, we aimed to improve a method to obtain active phage by transferring of environmental DNA into bacteria. The occurrence of active unknown bacteriophages after the transformation of *E*. *coli* with DNA extracted from the environmental source was tested. Moreover, the method can be innovative method for the discovery of novel phages from different sources.

## Materials and methods

### a) Total DNA isolation

Sampling and DNA isolation were done as described in our previous study [18]. Briefly, the wastewater sample was collected under sterile conditions from sewage of Hospital of Aydin Adnan Menderes University. Wastewater sample (100ml) were centrifugated (5 min at 5000 rpm) and supernatant was used for DNA extraction. The supernatant was filtered with 0.22 μm filter to remove bacteria and other higher microorganisms and centrifuged at high speed (15,000rpm) for 3 hours. Supernatant was discarded and pellet was resuspended in 100 μl of Saline-Magnesium buffer (SM buffer:100 mM NaCl, 8 mM MgSO_4_•7H_2_O, 50 mM Tris-HCl and 0,02% gelatine).

For DNA isolation from hospital wastewater and phage culture, the same protocol was used as described above [18]. DNAse I (Geneaid, Taiwan) to a final concentration of 1 μg/ml was added to the filtered wastewater sample/the concentrated phage suspension from culture and incubated at 37°C for 30 minutes. The DNAse I was inactivated at 70°C for 10 minutes. Then, Proteinase-K (BioShop, Canada) to a final concentration of 0.1 mg/ml was added to DNAse treated phage suspension and was incubated at 56°C for an hour to degrade viral capsid proteins. Subsequently, DNA was purified by using phenol:chloroform:isoamyl alcohol (25:24:1). Concisely, an equal volume of phenol:chloroform:isoamyl alcohol was added into the DNA containing solution, mixed by vortexing and was centrifugated at 13,000 rpm for 5 minutes at room temperature. The aqueous phase that contains DNA was transferred into a new tube, and 1/10 volume of 3 M Sodium acetate (pH:5.2) was added and mixed. Equal volume of isopropanol was added into DNA solution, mixed and incubated at -20˚C for an hour. After incubation, it was centrifugated at 15,000 rpm for 30 minutes at 4°C. The supernatant was discarded, and the pellet was washed with 300 μl of 70% ethanol. It was centrifugated again at 15,000 rpm for 5 minutes at 4°C, and the supernatant was discarded. The pellet was dried and resuspended in 50 μl of distilled water. A volume of 5 μl of DNA solution was run on 1% agarose gel and the concentration of the isolated DNA was measured by Nabi UV/Vis Nano Spectrophotometer (MicroDigital, South Korea).

### b) Transformation of *E*. *coli* DH10B and *E*. *coli* BL21

#### i) Preparation of chemically competent *E*. *coli* cells and bacterial transformation

For preparation of chemically competent *E*. *coli* DH10B and *E*. *coli* BL21 cells, “Preparation and Transformation of Competent *E*. *coli* Using Calcium Chloride” method was used with minor modifications [19]. All centrifugation steps were processed at +4˚C. Briefly, *E*. *coli* DH10B/BL21 culture at mid-log phase (OD_600_: 0.4–0.6) was incubated on ice for 30 minutes and centrifuged at 5.000 rpm for 10 min. The pellet was washed with dH_2_O, 10% glycerol solution and 80 mM MgCl_2_-20 mM CaCI_2_ solution. After final centrifugation (5,000 rpm for 10 min), the pellet was resuspended in 0.1 M CaCI_2_ solution. A volume of 100 μl competent cells and 10 μl of phage DNA were mixed and incubated on ice for 30 minutes. For transformation, the mixture was incubated at 42˚C for 2 minutes and immediately transferred onto ice for 2 minutes. A volume of 900 μl of Tryptic Soy Broth (TSB) was added to cells and was incubated at 37˚C. After an hour, the cells were transferred into 10 ml of TSB and incubated overnight. Next day, the cells were centrifuged at 5,000 rpm for 10 min and the supernatant was filtered through 0.22 μm filter. The filtrate was used for continuous steps.

#### ii) Preparation of E. coli electrocompetent cells and bacterial transformation

For preparation of electrocompetent *E*. *coli* DH10B/BL21 cells, “Transformation of *E*. *coli* by Electroporation” method was used with major modification [20]. All centrifugation steps were processed at +4˚C. Briefly, *E*. *coli* DH10B/BL21 culture at mid-log phase was incubated on ice for 30 minutes and centrifuged (5,000 rpm for 10 min). The cells were washed with dH_2_O for 2 times and centrifuged (5,000 rpm for 10 min). Original protocol was slightly modified, and a washing step was added using 80 mM MgCl_2_-20 mM CaCl_2_ solution. The pellet was washed with 10% glycerol solution for twice and centrifuged (5,000 rpm for 10 min). The pellet was resuspended in 10% glycerol solution. A volume of 10 μl of the purified DNA and 100 μl of electrocompetent cells were mixed and hold on ice for 15 minutes. The mixture was transferred to 0.2 cm electroporation cuvette and electroporation was processed with Ec2 programme (2.49 kV-1 pulse) by using MicroPulser Electroporator device (Bio-rad laboratories, Inc, USA). A volume of 900 μl of Tryptic Soy Broth (TSB) was added to cells and was incubated at 37˚C. After an hour, the cells were transferred into 10 ml of TSB and incubated overnight at 37˚C. On the following day, the cells were centrifuged (5,000 rpm for 10 min), and the supernatant was filtered through 0.22 μm filter. The filtrate was used for continuous steps as source of phage.

### c) Investigation of transformation efficacy

#### i) Spot test

Spot test was used as described previously [21]. Shortly, tenfold serial dilutions of the source of phage were prepared using SM buffer from 10^0^ to 10^−8^. Then, 100 μl of logarithmic phase (OD_600_: 0.4–0.6) *E*. *coli* DH10B and *E*. *coli* BL21 bacterial culture was added to 5 ml of soft agar (0.5% w/v agar) at 45–50°C, vortexed and poured onto the top agar (1.5% w/v agar) and left to solidify. Following this step, 10 μl of the previously prepared phage dilutions were dropped onto the pre-marked areas of the agar plate. After the drops were absorbed, the petri dish was inverted and incubated at 37°C overnight. Next day, the lytic activities of phages were observed.

#### ii) Double-layer agar assay

The double-layer agar method was performed as previously described [22]. Briefly, 100 μl of the previously prepared phage dilutions and 100 μl of logarithmic phase *E*. *coli* DH10B/BL21 bacterial culture was added to 5 ml of soft agar (0.5% w/v agar) at 45–50°C and mixed. It was poured onto the top agar plate and incubated at 37°C overnight. Following, the lytic activities of the phages were observed by plaques formation.

### d) Purification and characterization of the transformed phage

#### Bacteriophage purification

The phage purification was performed with the previously described method [23]. Briefly, a phage plaque from double-layer agar assay was picked by pipet tip and inoculated into the logarithmic phase *E*. *coli* DH10B bacterial culture (OD_600_: 0.4–0.6) and incubated at 37°C overnight. Next, the culture was filtered through 0.22 μm filter. The purification was repeated by using the phage filtrate until obtaining homogenous plaque morphology on the agar plate. The phage filtrate was stored at 4°C in the dark. Additionally, the titer of the phage suspension was determined by the double-layer agar assay as described above.

#### Host range determination

The bacteria used to investigate host range of the phage were listed in Table 1. The host range of the transformed phage ADUt was tested against a total of 45 bacterial panels from 12 different genera and 24 different species, closely related to *E*. *coli* (*Salmonella*, *Shigella*, *Enterobacter*) and other pathogenic bacteria. Tryptic soy agar and tryptic soy broth media were used for culture of these bacteria, and they were incubated at 37°C overnight. Host range analysis was performed by using spot-test method as described above.

**Table 1:** The strains used in this study.

Bacteria	Strain ID	Lysis
*Acinetobacter* spp.	RDPRM 1836	0
*Acinetobacter* spp.	RDPRM 1423	0
*Acinetobacter* spp.	RDPRM 3281	0
*Bacillus cereus*	ATCC11778	0
*Bacillus coagulanse*	DSM1	0
*Bacillus sphaericus*	DSM396	0
*Bacillus subtilis*	ATCC6633	0
*Enterobacter spp*.	RDPRM 3060	0
*Enterobacter spp*.	RDPRM 365	0
*Enterococcus faecium*	RDPRM V585	0
*Enterococcus fecalis*	ATCC 51299	0
*Enterococcus faecalis*	ATCC 29212	0
*Enterococcus fecalis*	JH2-2	0
*Escherichia coli*	ATCC 43893	0
*Escherichia coli*	ATCC 35401	0
*Escherichia coli*	ATCC 35150	0
*Escherichia coli*	ATCC 25922	+1
*Escherichia coli*	BL21 (DE3)	+3
* *Escherichia coli*	K12-DH10B	+4
*Escherichia coli*	DH5α	+4
*Listeria innocua*	ATCC 33090	0
*Listeria monocytogenes*	ATCC 19111	0
*Micrococcus luteus*	ATCC 9341	0
*Pseudomonas aeruginosa*	ATCC 27583	0
*Pseudomonas aeruginosa*	ATCC 15692	0
*Pseudomonas aeruginosa*	DSM 22644	0
*Pseudomonas aeruginosa*	NCTC 6750	0
*Pseudomonas aeruginosa*	RDPRM 1126	0
*Pseudomonas aeruginosa*	RDPRM 1834	0
*Pseudomonas aeruginosa*	RDPRM 1897	0
*Pseudomonas aeruginosa*	RDPRM 399	0
*Pseudomonas putida*	ATCC 47054	0
*Salmonella abony*	NCTC 6017	0
*Salmonella enterica*	RSSK 760	0
*Salmonella enterica*	RSSK 96030	0
*Salmonella enteritidis*	RSSK 91	0
*Salmonella typhimurium*	RSSK 95091	0
*Shigella boydii*	RSSK 1040	0
*Shigella dysenteriae*	RSSK 4070	+4
*Shigella flexneri*	RSSK 6024	+2
*Shigella sonnei*	RSSK 96021	0
*Staphylococcus aureus*	ATCC 43300	0
*Staphylococcus aureus*	ATCC 29213	0
*Staphylococcus aureus*	RN4220	0
*Vibrio parahaemolyticus*	NCTC10903	0

* Was used as indicator host in this study. +4: complete clearing, +3 clearing throughout but with faintly hazy background, +2 substantial turbidity throughout the cleared zone, +1 a few individual plaques, 0 no clearing.

#### Sequencing and bioinformatics analysis of Escherichia phage ADUt genome

Phage sequence was performed by Genera Inc, Türkiye. The libraries were prepared from isolated samples using the Nextera DNA Prep Library Prep Kit (Illumina, San Diego, CA) and sequenced on Illumina Nextseq 500 (Illumina, USA) platform with a 2x150 cycle. The quality of the raw data was evaluated using FastQC v.0.11.5 (Babraham Bioinformatics) and low-quality bases, primers, and remnant adapters were trimmed using Trimmomatic v.0.32 with default parameters [24]. The reads were assembled by using MEGAHIT v1.2.9 with default parameters [25]. To investigate genome similarity of the phage against Genbank database, BLASTN analysis was carried out.

#### Determination of restriction profile of the phage ADUt

DNA isolation from the phage obtained by transformation, ADUt, was performed as described above. The isolated bacteriophage DNA was measured by Nabi UV/Vis Nano Spectrophotometer (MicroDigital, South Korea) and run on 1% agarose gel. DNA was restricted at 37°C for 30 minutes with different restriction enzymes as recommended by the manufacturer (Thermo scientific, USA); PstI, BamHI, NotI, XbaI, DpnI, MvaI, LguI, NlaIII, MspI, NheI, PvuII, MunI, PaeI, SacII, XapI, FspBI, HincII, SfiI, TatI, TruI, EcoRI, HindIII, SalI, SacI, KpnI, SmaI, DpnI, NdeI, BglII and HinfI. The restriction products were run on the 1% agarose gel. The in-silico analysis performed with DNASTAR software (DNASTAR, Inc., USA)

#### Phylogenetic analysis

To construct phylogenetic tree VipTree 3.3 tool which generates proteomic tree of the phage genome sequence was used [26]. The reference genomes including prokaryotic dsDNA viruses were included for comparison.

#### Genome availability

The genome sequence of Escherichia phage ADUt has been deposited in GenBank under the following accession number: OQ466432.

## Results

### From environmental DNA to phage

**Fig 1:**
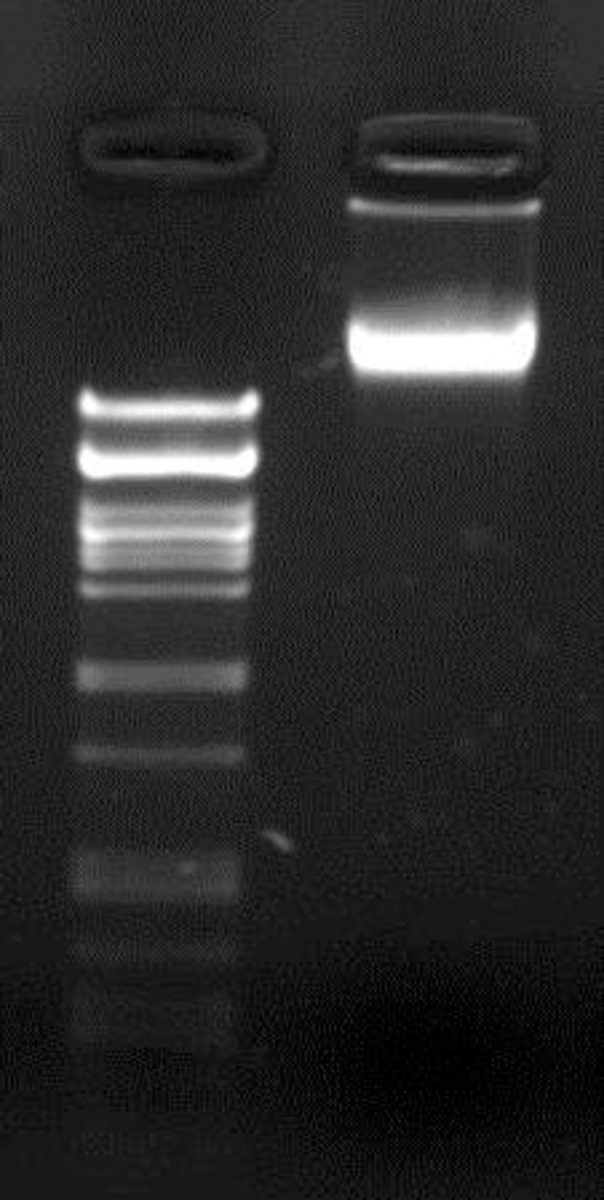
Environmental DNA extracted from the hospital wastewater. Left: Lambda-PstI marker, right: total phagome DNA (30 ng/μl concentration).

**Fig 2:**
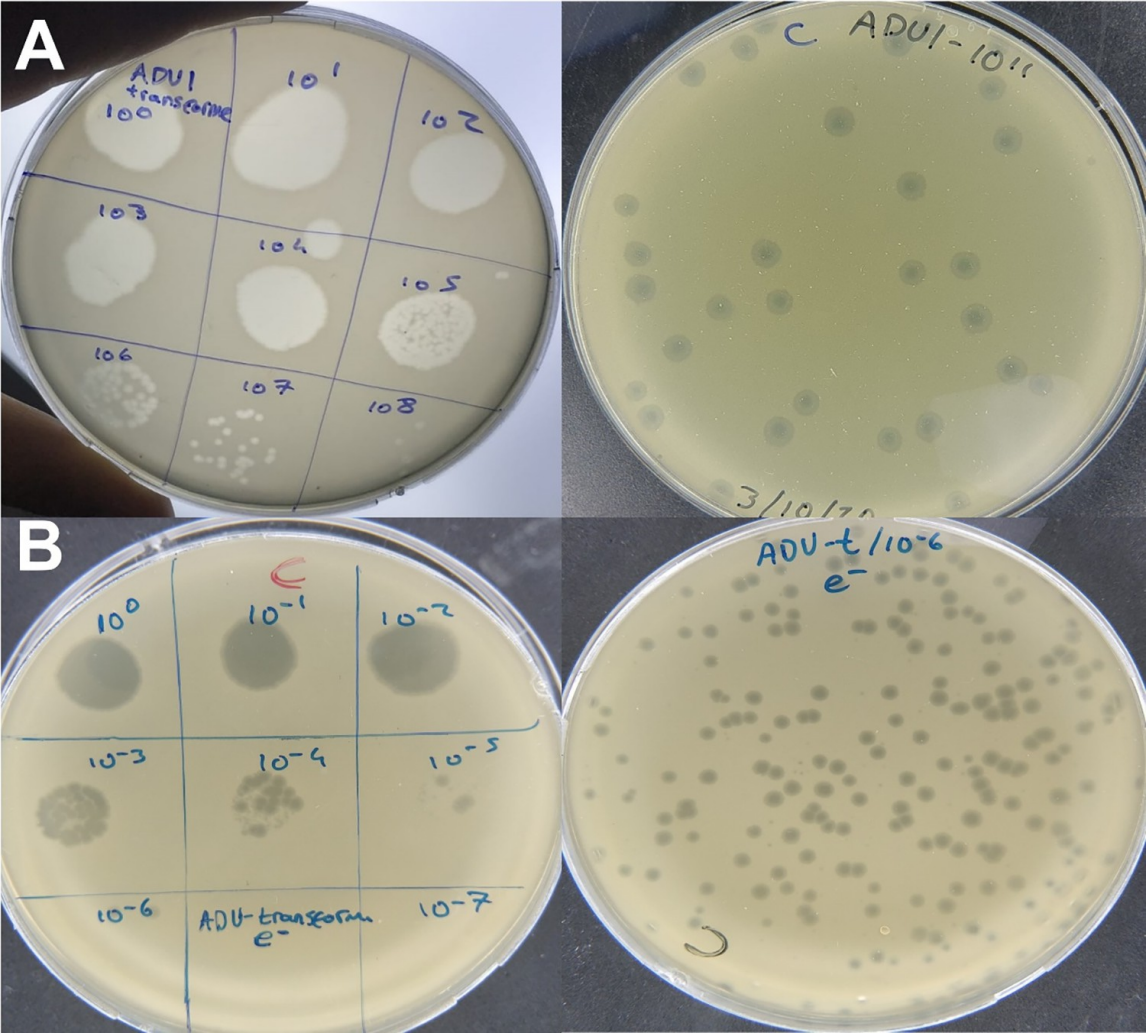
Phage plaque formation after transformation of environmental DNA extracted from wastewater into chemical competent and electrocompetent cells. **A)** Phage plaques obtained from transformation of chemical competent cells. Left; Agar spot method, right; Double-layer agar method. The titer of phage after transformation is approximately 10^12^ pfu/ml. **B)** Phage plaques obtained from transformation of electrocompetent cells. Left; Agar spot method, right; Double-agar plating method. The titer of phage after transformation is approximately 10^8^ pfu/ml.

The isolated environmental total DNA from hospital sewage is shown in Fig 1. The purified DNA was used to transform *E*. *coli* DH10B and *E*. *coli* BL21 by two different transformation methods, electroporation and chemical transformation. After transformation, phage plaques were observed only for *E*. *coli* DH10B containing plates, and not for *E*. *coli* BL21. The transformation efficacies were evaluated with agar spot test and double-layer assay. While the phage titer was calculated as 10^12^ pfu/ml for the chemical transformation method (Fig 2A), the titer was calculated as 10^8^ pfu/ml for the electroporation method (Fig 2B). The chemical transformation method gave more successful results for the transfer of phage DNA to the host. Since the plaques formed had similar morphology, one plaque was chosen for further studies and named as *Escherichia* phage ADUt.

### Re-transformation of *Escherichia coli* with ADUt phage DNA

**Fig 3:**
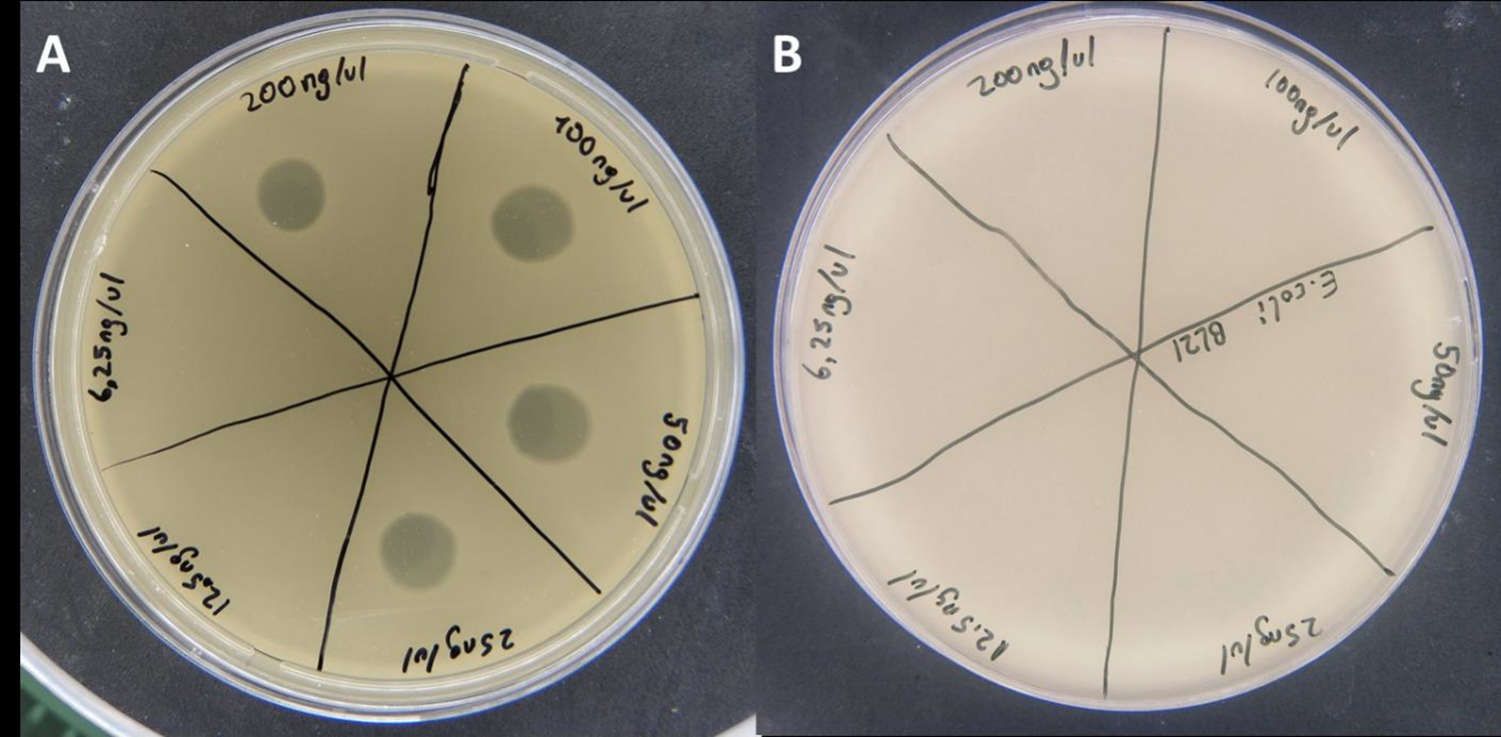
Re-transformation of ADUt phage DNA into *E*. *coli* DH10B and *E*. *coli* BL21 strains. **A)** Phage production was yielded by transformation with DNA at 25, 50, 100 and 200 ng/μl concentrations in *E*. *coli* DH10B. **B)** Phage production did not yielded transformation with DNA at any concentrations in *E*. *coli* BL21.

*Escherichia* phage ADUt isolated from plaque obtained from the transformation of environmental DNA into *E*. *coli* DH10B was propagated until its titer reaches 10^10^ pfu/ml. The total DNA of the ADUt phage was isolated and DNA concentration was measured by Nabi UV/Vis Nano Spectrophotometer (MicroDigital, South Korea). Several concentrations of phage DNA were used, 6.25, 12.50, 25, 50, 100 and 200 ng/μl, for re-transformation. ADUt DNA was transferred to both *E*. *coli* DH10B and *E*. *coli* BL21 by chemical transformation. Phage plaques were observed with spot test after transformation of *E*. *coli* DH10B at 25, 50, 100 and 200 ng/μl concentrations, but no yield occurred with lower concentrations (≤12.5 ng/μl) (Fig 3). No plaque formation was observed after transformation of *E*. *coli* BL21 with all DNA concentrations used.

### Host range determination

The host range of the transformed phage ADUt was determined using a total of 45 bacterial strains from 12 different genera and 24 different species; 3 clinical *Acinetobacter spp*., 1 *Bacillus cereus*, 1 *Bacillus coagulanse*, 1 *Bacillus sphaericus*, 1 *Bacillus subtilis*, 2 *Enterobacter spp*., 1 *Enterococcus faecium*, 3 *Enterococcus fecalis*, 7 *Escherichia coli strains*, 1 *Listeria innocua*, 1 *Listeria monocytogenes*, 1 *Micrococcus luteus*, 8 *Pseudomonas aeruginosa*, 1 *Pseudomonas putida*, 1 *Salmonella abony*, 2 *Salmonella enterica*, 1 *Salmonella enteritidis*, 1 *Salmonella typhimurium*, 1 *Shigella boydii*, 1 *Shigella dysenteriae*, 1 *Shigella flexneri*, 1 *Shigella sonnei*, 3 *Staphylococcus aureus* and 1 *Vibrio parahaemolyticus*. To assess the success of infection by the ADUt phage, spot-test was used. It was determined that the phage ADUt infect only 3 species, *Escherichia coli*, *Shigella dysenteriae* and *Shigella flexneri*. Of the 45 strains tested, 6 were susceptible to different levels of ADUt phage infection (Table 1). The result showed the highest lytic activity against *Escherichia coli* DH10B which is indicator strain in this study, followed by *E*. *coli* DH5α and *Shigella dysenteriae*. It had weaker lytic activity against *E*. *coli* BL21, *Shigella flexneri* and *E*. *coli* ATCC 25922.

### Genome sequencing of ADUt phage

The sequencing of the ADUt genome was performed with NGS. The paired end library includes 136660 reads and average length of reads are 152 bp. FastQc report of the library showed a total of 133059 read pairs passed without poor quality. The whole genome of ADUt phage was assembled with MEGAHIT v1.2.9. The size of the genome was found to be 166 904 bp by genome assembly. The GC rate of the genome was 35.67%. It was found that the most similar phage to the ADUt phage was the Shigella phage Sf22 (NC_042039.1) by BLASTN analysis and the ratio was 96.88%.

### Restriction profiles of ADUt genome

**Fig 4:**
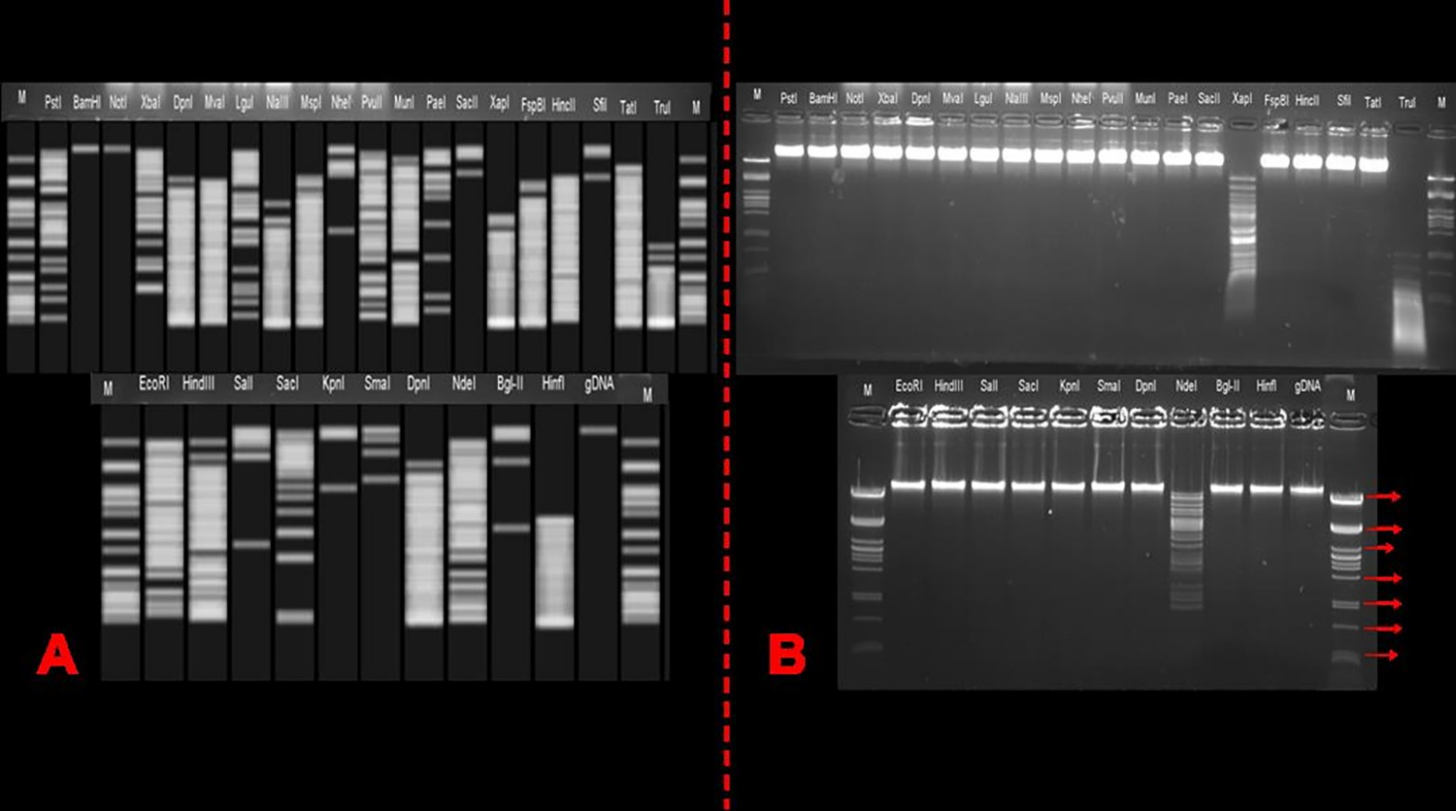
The restriction profile of the transformed phage ADUt with different restriction enzymes. **A)** The expected band size of ADUt phage with different restriction enzymes was obtained by *in silico* analysis of ADUt phage genome. **B)** The restriction profile of ADUt phage with different restriction enzymes was obtained by *in vitro* analysis of isolated ADUt phage DNA. This figure show that most of the enzymes except for XapI, TruI and NdeI were not able to DNA fragmentation probably due to resistance. M: Lambda-PstI marker. Top of gel-wells: The enzymes used for the restriction profile.

The restriction profile of the ADUt phage was determined both *in silico* and *in vitro* by using purified phage DNA and whole genome sequence of ADUt phage, respectively. The simulated gel images were showed presence of restriction sited for enzymes; PstI, XbaI, DpnI, MvaI, LguI, NlaIII, MspI, NheI, PvuI, MunI, PaeI, SacII, FspBI, HncII, SfiI, TatI, EcoRI, HindIII, SalI, SacI, SmaI, DpnI, BgLII, HinfI and KpnI (Fig 4). However, restriction of purified DNA using the same enzymes did not yield expected fragments *in vitro* that were generated *in silico*. For example, the formation of 39 DNA fragments in various sizes (between 42–11973 bp) was expected with PstI enzyme restriction according to *in silico* analysis. However, the DNA was not restricted by PstI enzyme and only phage genomic DNA band appeared on the agarose gel. In other words, the phage DNA was found to be resistant to these enzymes. NotI enzyme is non-cutter for the phage genome because the genome does not carry enzyme recognition site for NotI. BamHI enzyme is a one-cutter for the phage genome as in *in-silico* analysis. No fragments may be observed after restriction with BamHI which may be due to generation of large fragments that cannot be distinguished on the agarose gel (1%) electrophoresis used. Finally, cleavage with XapI, TruI and NdeI enzymes yielded similar band profiles in both the simulated gel and the agarose gel (Fig 4). For example, DNA fragments occurred in various sizes with restriction by NdeI enzyme for both *in silico* and *in vitro* experiments.

### Phylogenetic analysis

**Fig 5:**
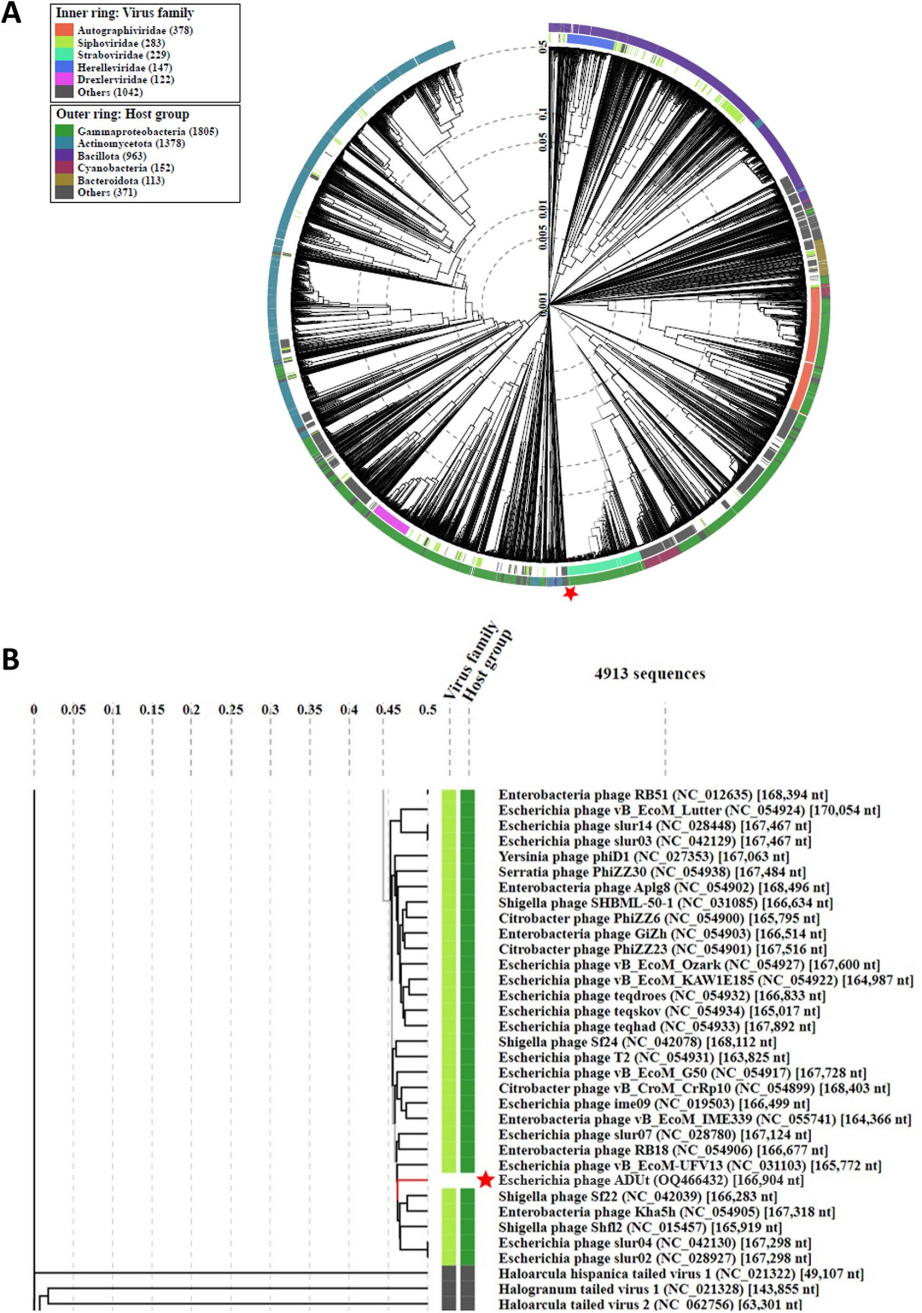
VipTree analysis of Escherichia phage ADUt with the reference phages. (A) ADUt was represented with star. The outer ring indicates hosts of the phages, and the inner ring indicates the family to which the phages belong. (B) The taxonomic relationship for ADUt and its relatives showed that ADUt phage belongs to the family *Straboviridae*, the subfamily *Tevenvirinae* and genus of *Tequatrovirus*. The closest relatives to ADUt phage are phages which infect *Escherichia* and *Shigella* genera.

Taxonomic analysis was performed with ViPTree server. This programme uses the proteomic tree based on genome-wide comparison with the reference genomes including prokaryotic dsDNA viruses in the database. Circular and rectangular trees were generated (Fig 5). The position of ADUt in the phylogenic tree indicated that ADUt is a member of *Straboviridae* family and *Tequatrovirus* genus. Phylogenetic tree demonstrated that the closest relatives of ADUt phage are phages which infect *Escherichia* and *Shigella* genera (Escherichia phage slur02, Escherichia phage slur04, Shigella phage Shfl2 and Shigella phage Sf22). However, ADUt phage is located in a different clade. The phylogenetic analysis showed ADUt phage was located in a different clade from its relatives. It can be explained by proteomic differences between ADUt phage and its relatives because VipTree analysis give opportunity to construct the tree.

## Discussion

Bacteriophages are the most abundant entities of the living world. As far as we know, our study is the first study in which phage could be obtained by transferring total environmental DNA into a living cell. It is well known that phages may lose their infectivity in the environment due to structural damages including breaking of tail protein or unfavorable conditions [27]. The use of DNA, which is more resistant than structural integrity of bacteriophages to the environmental stress, is an alternative method for bacteriophage isolation.

Environmental sources are rich in various phages due to allow the existance of their hosts. They can be containing even uncultured hosts and thereby their phages. The several environmental samples like wastewater, marine water, soil etc. draw attention as a phage source. DNA isolated from them can give chance to obtain novel phages or having unique properties. Although the environmental DNA is found to be productive this improved method, the isolation of DNA from environmental samples can be challenging due to substances in the samples or seasonal changes [28].

The traditional phage isolation techniques, such as plaque assays or filtration techniques are confidential and commonly used [15]. The methods are convenient to determine infectivity of plaque forming capability of phages. However, these methods have limitations to obtain phages from environmental samples because the environmental conditions may disrupt the phage integrity and hence infectivity [27]. So, these methods cannot yield any plaques and may not help to detect the phages even they exist in the sample. DNA-based phage detection methods have significant advantages to find out novel and endemic phages especially from environmental samples. Metagenomic analysis which is one of the DNA based phage detection, facilitates the detection of novel phages in the environmental sample [29]. This method relies on directly sequencing of DNA isolated from the sample. So, the integrity/infectivity of the phage is not important for this method. Solely, the multiplication of phages cannot be viable and their biologic characterization cannot be done. The improved method used in this study enables the detection as well as genomic and biologic characterization of the phages. However, DNA based phage detection methods have some limitations. There should be a minimum of DNA concentration for the method to be successful. On the other hand, the phages can be detected even with low phage titer by traditional methods. Consequently, our improved method used in this study has some advantages and limitations as metagenomic and traditional methods. The method to be used may be chosen subject to the isolation purposes like phage therapy, environmental monitoring etc. and laboratory sources.

Several methods have been developed for the phage DNA transformation. Electroporation [30], Calcium chloride (CaCl_2_) transformation [31] and *in vitro* packaging of phage DNA [32] are examples of these methods. The procedures include transformation of known phage DNA isolated from pure phage culture. To our knowledge, this is the first study in which environmental DNA is transferred to bacteria and an unknown phage is acquired. In this study, the modified electroporation method and the heat-shock method with CaCl_2_ were used and their success were compared. The CaCl_2_ method was found to be more successful. It is known that divalent ions such as Ca^+2^ and Mg^+2^ facilitate phage adsorption to the host [33]. For this reason, it is estimated that the presence of CaCl_2_, which is used in the competent cell preparation process, helps adsorption of the progeny phages during incubation and which increases success of transformation.

Environmental DNA was transferred into *E*. *coli* DH10B and *E*. *coli* BL21 strains. Active phage was yielded with transformation of *E*. *coli* DH10B. However, plaque formation was not observed after both the double layer agar and spot test when the transformation protocol was applied to the BL21 strain. On the other hand, phage plaque formation was again not observed after re-transformation of *E*. *coli* BL21. There are two possible explanations to this case. Firstly, the phage DNA is transferred to the *E*. *coli* BL21 strain, but the produced progenies are not capable of infecting the BL21 strain (lack of a receptor to attach to, etc.). However, in the host-range study of ADUt phage, it was shown that the ADUt phage was effective against the BL21 strain, so this possibility is invalid. Secondly, it is predicted that ADUt phage DNA is more protected than naked DNA when it is packaged. When genomic properties of *E*. *coli* DH10B and *E*. *coli* BL21 strains in terms of defense against foreign DNA were investigated, they were found different. McrA and McrBC, which are restriction endonucleases responsible for breaking down foreign DNA entering the cell, are encoded in the BL21 genome, while these genes are not found in the DH10B genome [34]. Therefore, it is likely that naked phage DNA entering the cell was removed by BL21 endonucleases not DH10B. This assumption needs further investigation. From this point of view, it is estimated that the success will be higher if the hosts that are sensitive to phages, that is, those with a weak defense system against foreign DNA, are preferred for this method. Several concentrations of the purified ADUt phage DNA were used for the transformation of *E*. *coli* DH10B, however only high concentrations 25, 50, 100 and 200 ng/μl granted occurrences of phages. No phage zone was observed at 6.25 and 12.50 ng/μl, which indicates that there is a minimum concentration of DNA for a successful transformation.

Monovalent phages inhibit the growth of a single specific species [35], however polyvalent phages with a wide host range can infect different genera or species. The monovalent and polyvalent phages have advantages depending on the aim. If a specific species was targeted, the use of monovalent phage is more preferrable. For example, an infection may be treated with monovalent phage without disturbing body flora. The polyvalent phages may be preferred to destroy bacteria in food or wastewater [35]. Our host range study indicated that ADUt phage infects *S*. *flexneri*, *S*. *dysenteriae*, and *E*. *coli*. Consequently, it is a narrow-spectrum polyvalent phage. *Doore et al*. isolated several phages that are able to infect two different genera specific to *E*. *coli* and *Shigella* [36]. Also, it was reported that HY01, SFP10, PS5 and vB_EcoM_swi3 phages can infect both *E*. *coli* and *Salmonella* [37–40]. It has been shown that phage KFS-EC3 can infect three different genera, including *E*. *coli*, *Shigella* and *Salmonella* [35]. The phylogenetic analysis showed that ADUt phage is relative of phages assigned as *Shigella* and *Escherichia* phages. Possibly, the phages are polyvalent phages and can infect both *Shigella* and *Escherichia* hosts. However, they were named with their indicator strain. Sf22 phage which is the highest genome similarity (96.88%) to ADUt phage is effective against many *Shigella* strains but not effective against *E*. *coli* strains [36].

Environmental DNA may be an important source of new phage acquiring. Our study showed that DNA transformation yielded active bacteriophage with both chemical transformation and electroporation. The selection of host is a significant point to obtain active phage. The host which has a weaker defense mechanism against foreign DNA should be preferred.

## Conclusions

A small fraction of all phages in the world has been discovered and investigated so far. The use of only conventional methods (agar spot-test, double-layer agar assay) to discover new phages is associated with due to limited the number of phages discovered. The development of new techniques is needed for obtaining new phages from different sources, especially the environment. It was the first time to obtain active phage from environmental DNA with both chemical transformation and electroporation in this study. Genome sequencing and host range analysis were performed for the characterization of the new phage obtained. The results of the present study show evidence for the use of DNA transformation to obtain new bacteriophages.
